# The Novel Role of the Expression of Toll-like Receptors TLR-5, TLR-6, and TLR-9 and Associated Up-Regulation of Programmed Cell Death 1 Receptor (PD-1) and Its Ligand (PD-L1) in Lung Sepsis

**DOI:** 10.3390/ijms26052274

**Published:** 2025-03-04

**Authors:** Georgios Sinos, Dimitrios Schizas, Alkistis Kapelouzou, Maximos Frountzas, Michalis Katsimpoulas, Konstantinos S. Mylonas, Emmanouil I. Kapetanakis, Alexandros Papalampros, Theodore Liakakos, Andreas Alexandrou

**Affiliations:** 1 First Department of Surgery, National and Kapodistrian University of Athens, “Laikon” General Hospital, 115 27 Athens, Greece; schizasad@gmail.com (D.S.); a_papalampros@hotmail.com (A.P.); theodlia@hotmail.com (T.L.); alexandrouandrea@hotmail.com (A.A.); 2 Center for Clinical, Experimental Surgery and Translational Research, Biomedical Research Foundation of the Academy of Athens, 115 27 Athens, Greece; akapel@bioacademy.gr (A.K.); mkatsiboulas@bioacademy.gr (M.K.); 3 First Propaedeutic Department of Surgery, National and Kapodistrian University of Athens, “Hippocration” General Hospital, 115 27 Athens, Greece; froumax@hotmail.com; 4 Department of Cardiac Surgery, Onassis Cardiac Surgery Center, 176 74 Athens, Greece; ksmylonas@gmail.com; 5 Department of Thoracic Surgery, National and Kapodistrian University of Athens, “Attikon” University Hospital, 124 62 Athens, Greece; emmanouil.kapetanakis@gmail.com

**Keywords:** TLR, lung, PD-L1, sepsis, experimental

## Abstract

Sepsis is a leading cause of death in hospitalized patients. The underlying pathophysiologic mechanisms of sepsis have not been fully elucidated thus far. The receptor of programmed cell death 1 (PD-1) and its ligand (PD-L1), in combination with the Toll-like receptors (TLRs), seem to contribute considerably in systematic responses during sepsis. Investigating the relationship between them and identifying potential target pathways is important in the future management of sepsis, especially in relation to acute lung injury. This study investigated the interactions between TLR-5, -6, and -9 and PD-1/PD-L1 expression in a septic mouse model. Sixty C57BL/6J mice were included and categorized in six study groups. Three sepsis (S) groups (24 h, 48 h, and 72 h) and three sham (Sh) groups (24 h, 48 h, and 72 h) were created. Cecal ligation and puncture (CLP) was utilized to simulate sepsis in the S groups. Hematological analysis and lung tissue histopathological analysis were performed after 24 h, 48 h, and 72 h. Significant decreases in S groups compared to Sh groups in WBC and lymphocyte counts at 24, 48, and 72 h were observed. Significant increases in S groups compared to Sh groups in RBC and monocyte counts, IL-6 and IL-10 levels, alveolar flooding, and alveolar collapse were demonstrated by histopathological analysis. This study suggested a strong correlation between TLR expression and PD-1/PD-L1 up-regulation in lung tissue during sepsis. These molecules, also, seem to contribute to the histopathological changes in lung tissue during sepsis, leading to acute lung injury.

## 1. Introduction

Sepsis is an overwhelming inflammatory response syndrome secondary to infection, which affects millions of people and is a major cause of death for patients hospitalized in intensive care units (ICUs) [1]. Almost 25% of patients diagnosed with sepsis eventually die [2,3,4]. Furthermore, sepsis seems to be a major therapeutic challenge as no improvements in patient mortality has been reported despite developments in antibiotic therapies and protocols [5,6]. In fact, no targeted therapy for the underlying pathophysiology of sepsis has been discovered while the healthcare costs for treating these patients in developed countries, for example in the United States, range from USD 16.7 billion to USD 24.3 billion [7,8].

Pattern recognition receptors (PRRs), and especially Toll-like-receptors (TLRs), have a major impact in innate immune response through the production of cytokines, interferons, and nucleotides. Their discovery has provided a significant level of information and has helped clinicians and bio scientists in understanding the molecular pathways between inflammation, innate immunity, and various pathologies [9,10]. Currently they are considered crucial receptors for the initiation of the inflammatory response in sepsis, and it is hoped that in the future, they may constitute novel and important therapeutic targets for sepsis [9,10]. In addition, myeloid differentiation primary response 88 (MyD88) is a crucial signaling inflammatory pathway that involves the activation of nuclear factor-κB (NF-κB) and Mitogen-activated protein kinase (MAPK) [9,11,12,13]. The expression of TLRs and mass release of inflammatory mediators through the activation of NF-κB and MyD88 are involved in the pathophysiology of sepsis and in multiple organ dysfunction syndrome (MODS) [14,15]. In particular, TLR-5 and -6 using the MyD88 protein induce NF-κB activation, resulting in the release of inflammatory cytokines. TLR-9 also seems to contribute to the immune response through the activation of MyD88 and NF-κB, contributing to the mass release of cytokines from dendritic cells (DCs) [16]. It is known that flagellin stimulates TLR-5 up-regulation and its stimulation contributes to colonic inflammation [17]. In addition, TLR-6 seems to have a strong correlation with the immune response against bacteria and the progression of inflammatory processes. Furthermore, the degradation of TLR-6 seems to attenuate the inflammatory response [18]. Both B and T cells are sensitive to TLR signaling with T cells, responding to many TLR stimuli including those of TLR-2, -3, -4, -5, -7, and -8 [19]. Similarly B cells have been demonstrated to have a distinct pattern of expression of many TLRs such as -1, -2, -4, -6, -7, and -9 [20].

Programmed cell death 1 (PD-1) is a co-inhibitory receptor that negatively regulates immune responses. PD-1 is expressed in T cells, B cells, and monocytes, which can differentiate into macrophages, natural killer cells, and dendritic cells. PD-L1 and PD-L2 are the two known ligands of PD-1. PD-L1 is, also, expressed in B and T cells, as well as in many solid organs including the lung, liver, and pancreas [21]. PD-1 and PD-L1 mediate the exhaustion of T cells in sepsis [22,23,24]. An up-regulation of PD-1, PD-L1, and PD-L2 in the CD4+ cells on days 1–2 and 3–5 after the onset of septic shock has previously been observed and reported [25]. Moreover, the PD-1/PD-L1 pathway seems to contribute directly to T-cell exhaustion and a lack of viral control during chronic infections [26].

Evidence suggests a correlation between TLRs and PD-1/PD-L1 expression through the MyD88 dependent pathways. The study of these pathways has been a major focus for sepsis specialists [12,21,22,23,24,25]. It has been shown that the expression of PD-1 after TLR-2 receptor activation is completely blocked in MyD88^−/−^ cells [27]. The increased expression of PD-L1 following the activation of TLR-3 in neuroblastoma cells and TLR-4 in colonic mucosal fibroblasts has been also described [28,29]. It may thus be hypothesized that TLR-mediated PD-1 induction could be the pathway through which bacteria and other pathogens induce the inhibition of the immune response. Again, this pathway could constitute a potential therapeutic target for sepsis. It is well known that the respiratory system is significantly affected in sepsis and that it is often the primary organ to fail [30]. Because of systemic inflammation, the lungs can suffer profound cell infiltration, non-cardiogenic pulmonary edema, and diffuse alveolar damage, which eventually can lead to significant respiratory failure [30,31].

Acute lung injury (ALI) and acute respiratory distress syndrome (ARDS) are two life-threatening conditions that can occur in septic patients [30]. However, a complete picture of the mechanistic role of bacterial peritonitis-induced sepsis in lung pathology is still unclear and under-investigated. Considering their potential as therapeutic targets, the elucidation of the relationship of TLRs (especially the less-well-known -5, -6, and -9) with the PD-1/PD-L1 system is imperative. The aim of this study was to investigate the expression of TLR-5, -6, and -9 and their binding molecules (MyD88, IRAK, TIRAP, and NFkB), and their relation to the PD-1/PD-L1 system, in the pulmonary tissue of a septic mouse model.

## 2. Results

### 2.1. Hematological Analysis

**Figure 1:**
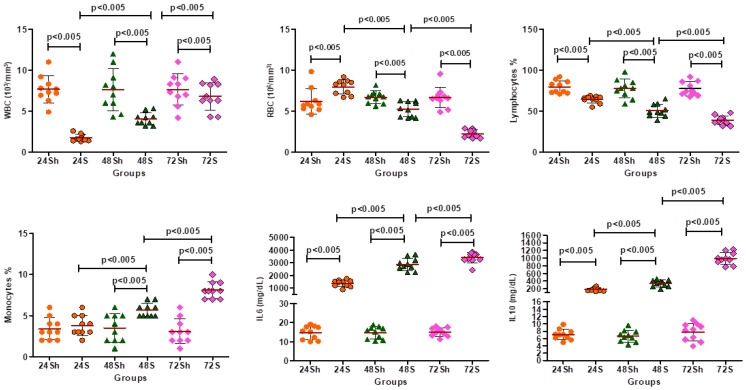
Scatter plot diagram of the blood analysis demonstrating specific groupings in experimental time periods/hours. Statistically significant differences between experimental groups are presented with *p* < 0.05.

A significant decrease was demonstrated in the S groups compared to the Sh groups in WBC and LY counts at 24, 48, and 72 h. This is not something unexpected in sepsis. On the other hand, a significant increase was revealed in S groups compared to Sh groups in RBCs (24, 48, and 72 h), MOs (48 and 72 h), IL-6 (24, 48, and 72 h), and IL-10 (24, 48, and 72 h). Statistically significant differences were, also, observed in WBCs, RBCs, LYs, MOs, IL-6, and IL-10 between 24 and 48 h and between 48 and 72 h among the S groups (Table 1, Figure 1). Again, this is something that has been established in sepsis as, through time, there is an increase in the presence of inflammatory markers and a decrease in the number of immune cells through consumption.

### 2.2. Histopathological Analysis

**Figure 2:**
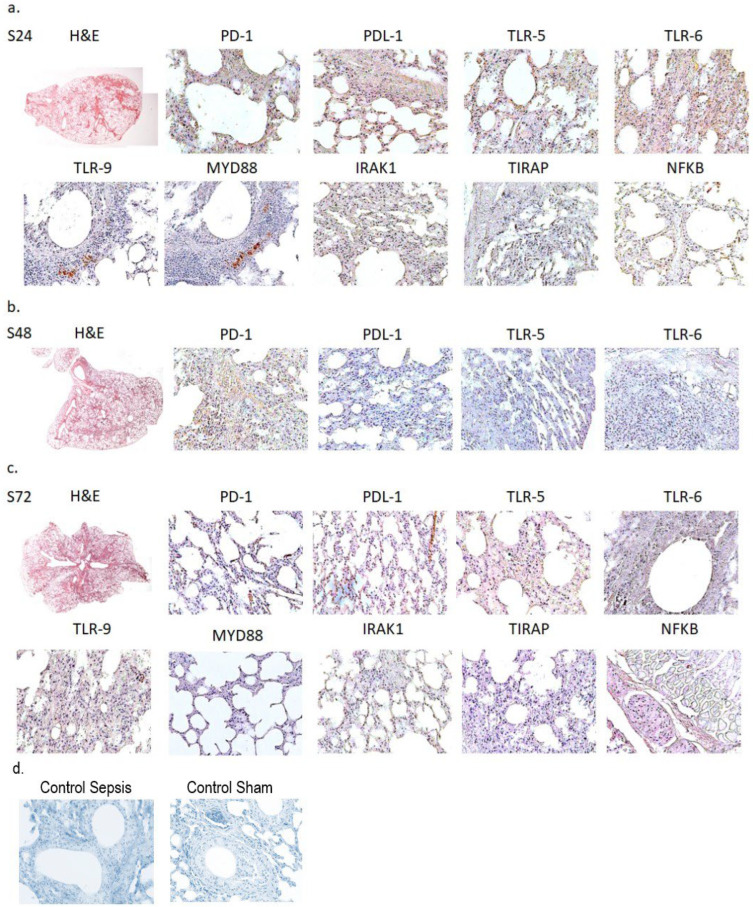
Lung tissue specimens stained with hematoxylin and eosin, demonstrating immunohistochemical reaction. Images (×1.5) from lung sections are shown at 24 h (**a**), 48 h (**b**), and 72 h (**c**) in the septic groups. Controls for sepsis and sham groups (**d**) are also shown. Original magnification was ×40. Images are representative of 10 animals.

Lung tissues from all experimental groups were examined, and the immunohistochemistry analysis of PD-1; PD L-1; TLRs-5, -6, and -9, MyD88; IRAK1; TIRAP; and NFkB was performed. The immunohistochemistry of PD-1; PD L-1; TLRs-5, -6, and -9; MyD88; IRAK1; TIRAP; and NFkB demonstrated edema and alveolar damage in S24 and S48 groups, which was compatible with a septic response. In addition, alveolar collapse was observed in the S72 group, which, again, was not unexpected as ALI set in. Figure 2a–c show the most representative images of the histopathology analysis in the S groups, clearly demonstrating the stages of ALI and ARDS mentioned above. Alveolar flooding and alveolar collapse were noticed in the S groups after the first 24 h of being in a septic condition. In addition, the marked sepsis-induced expression of TLRs, PD-1, and PD-L1 was revealed, and this was an expected response. Positive cells in the lungs of septic mice were stained with a brown color while cell nuclei were stained blue.

In addition, a computer-assisted histochemistry quantification analysis of the S group was performed and is presented in Table 2. The table more clearly demonstrates the findings mentioned above.

### 2.3. TLRs and PD-1/PD-L1 Expression in Lung Tissue

**Figure 3:**
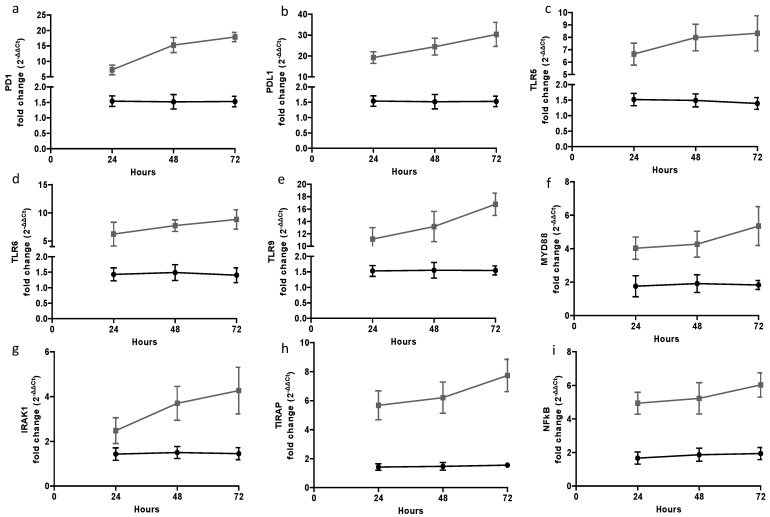
Linear graphs of gene expression at 24, 48, and 72 h utilizing qRT-PCR analysis in lung tissue specimens for (**a**) PD-1, (**b**) PDL-1, (**c**) TLR-5, (**d**) TLR-6, (**e**) TLR-9, (**f**) MYD88, (**g**) IRAK-1, (**h**) TIRAP, and (**i**) NFkB. The axes represents fold change over hours/period. The black line represents the sham groups and the gray line the septic groups.

The expression of PD-1; PD-L1; TLR-5, -6, and -9; MYD88; IRAK1; TIRAP; and NFkB was quantified by RT-PCR at 24, 48, and 72 h after CLP-induced sepsis (Figure 3). A significant mRNA up-regulation of all genes during sepsis when comparing S groups and Sh groups (*p* < 0.05) was demonstrated. Such an up-regulation constitutes part of the expected inflammatory response during sepsis, and our results were similar to previously reported ones. PD-1 and PDL-1 mRNA expression was increased during septic conditions, again not unexpectedly. PD-1 mRNA expression was higher in the S48 group compared to the S24 group and in the S72 group compared to the S48 group. This demonstrated the peak of activation occurring at the 48 h mark of an inflammatory response. In addition, PD-L1 mRNA expression was increased in septic animals, probably due to acute lung injury that had been induced by sepsis. Marked differences were, also, noted between the S24–S48 and S48–S72 groups.

TLR-5 and TLR-6 mRNA expression was not significantly different during septic progression, except for TLR-5 in the S24 vs. S48 groups and the S24 vs. S72 groups (Figure 3c). This was expected considering their similar roles in the induction pathway. However, the mRNA expression of TLR-9 was markedly increased after 24 h of sepsis. In fact, there was an unanticipated significant increase during septic progression, with higher expression in the S72 group followed by the S48 and S24 groups (Figure 3e). PCR analysis revealed the significant up-regulation of MyD88 and TIRAP during sepsis (Figure 3f,h). On the other hand, while the IRAK-1 and NFkB genes were up-regulated in septic animals, no statistical difference was found between the two animal groups, except for IRAK-1 between the S24 vs. S48 groups and the S24 vs. S72 groups.

### 2.4. Correlation Between mRNA Genes

PD-1 expression was significantly correlated with PDL-1 and IRAK-1 expression in the S24 group, which was expected considering their role in sepsis response. In addition, it was correlated with PDL-1, MYD88, and IRAK-1 in the S48 group (Table 3). PDL-1 was significantly correlated with MYD88 and IRAK-1 in the S24 group, as well as with TLR-6 and MYD88 in the S72 group. On the other hand, TLR-6 was negatively correlated with TLR-5 in the S24 group (Table 3). TLR-9 was negatively correlated with TLR-6 in the S48 group and positively correlated with NFkB in the S48 group. Finally, TLR-6 was significantly correlated with MYD88 in the S72 group and TLR-9 was significantly correlated with TIRAP in the S72 group.

## 3. Discussion

In this experimental study, bacterial peritonitis was induced using a CLP model, which has been considered as the “gold standard” for septic simulation in animal models aged 16–20 weeks and which more closely resembles septic conditions in the elderly [31,32,33]. This model has been shown to be reliable in investigating sepsis-induced ALI and ARDS [31,32,33].

It has previously been demonstrated that TLRs such as TLR-2, TLR-3, TLR-4, and TLR-7 are highly expressed in the lung tissues of septic mice, therefore suggesting a potential role in the pathogenesis of ARDS during sepsis [31]. The presence of a “distant effect phenomenon” has been proposed, where cecal puncture causes peritonitis and sepsis has an associated remote effect on the respiratory system [31]. Expanding upon these findings, this study additionally revealed an up-regulation of the TLR-5, -6, and -9 receptors during the progression of sepsis. This not only confirmed the already reported findings about the TLR-2, -3, -4, and -7 receptors but also revealed a previously unknown and unanticipated role for the TLR-5, -6, and -9 receptors. Specifically, TLR-5 and -6 were significantly up-regulated in our experimental model during the early stages of the septic condition while, more interestingly, TLR-9 was significantly increased during both the early and the late stages of septic response. This was quite a novel finding and highlighted an unexpected potentially protective role of TLR-9 during the early septic phases. Reduced levels of TLR-9 are associated with decreased immunopathological response early during sepsis, which could be correlated with the reduced mortality initially. However, once TLR-9 increases, the elevation of cytokines such as IL-6 and IL-10, to a lesser extent, occurs. In this study, however, IL-10 was elevated to similar levels compared to IL-6, which was unusual and certainly opens up a number of intriguing questions that require additional study.

In addition, the expression of TLR-9 in lung tissue was mainly induced after 48 h with a continuous upward trend, indicating a possible direct correlation with ongoing sepsis and acute lung injury. Previous studies had supported that TLR-9 expression was correlated to microbial and viral infections [34,35]. TLR-9 stimulates the production of pro-inflammatory factors such as IL-6 and participates in inflammatory immune responses, possibly through a TLR-9/MyD88-dependent pathway [36]. However, the implication of this late up-regulation of TLR-9 observed in this study and its significance is elusive and cannot directly be explained by the literature, constituting another novel and unexpected finding of the study. It has been established that early sepsis is mediated by the pathogenic TLR triggering of TLR-5/-6 while sustained sepsis is most likely mediated through TLR-9 via the activation of P38 MAPK, which has been considered to play a critical role in the release of inflammatory mediators in sepsis and the inactivation of Akt, which is a key negative regulator of the inflammatory response [37]. Certainly, this finding is intriguing and “food for thought”, with further investigation into it warranted as it could provide a possible future therapeutic target!

Conversely, it is well known that TLR-5 recognizes flagellin, which is a component of motile bacteria, and this mechanism has been studied as a biomarker predicting Systemic Inflammatory Response Syndrome (SIRS) [38]. Furthermore, the protective role of TLR-5 agonists in respiratory bacterial infections had been suggested in previous studies [39].

PD-1 expression was significantly increased in the lung tissues of the septic animals, suggesting a central role in the pathogenesis of acute lung injury (ALI) during sepsis. In addition, PD-L1 was also expressed in the lungs of septic animals, further implying the crucial role of these ligands in the progression of sepsis. The expression of PD-1, PD-L1, and TLR-9 genes was mainly induced after the second day of septic progression with a continuous upward trend. This up-regulation was accompanied by comparable histopathological changes in the lung specimens of the septic animals. Previous studies had indicated the inhibitory role of PD-1 during sepsis and its involvement in T cell “exhaustion”, a condition followed by the apoptosis of the exhausted T cells and an ineffective immune response [40,41]. Moreover, PD-1 seemed to be highly expressed in the circulating T cells of septic patients with acute lung injury, indicating an important role in sepsis and acute lung damage [42]. Finally, PD-1 expression in patients with acute lung injury induced by sepsis had been correlated to increased mortality rates [43]. All these findings may indicate a persistent immune suppression associated with a continuous PD-1/PD-L1 expression and a possible correlation between TLRs and PD-1/PD-L1 signaling.

A significant increase regarding the expression of IRAK1 was observed in the lungs of septic mice between 24 h and 48 h after the onset of sepsis. However, no significant differences were observed after 48 h, suggesting that IRAK1 is correlated with signaling in the early stages of sepsis. Furthermore, TIRAP was amplified in the septic groups and a significant difference was observed between 48 h and 72 h of the onset of sepsis. TIRAP contributes to the signaling pathways of TLRs and had been correlated with acute lung injury in previous studies [44]. TIRAP expression in the early stages of sepsis was in accordance with the amplification in the expression of TLR-9, indicating a possible correlation. In addition, TLR-9 signaling has been related to MyD88 recruitment, followed by the activation of NF-κB, leading to acute lung injury and acute respiratory distress syndrome (ARDS) through the secretion of cytokines, which was consistent with our findings suggesting that sepsis induces inflammation by the synthesis of NF-κΒ and pro-inflammatory cytokines [45].

Another significant and new finding of this study was the correlation of mRNA expression in different groups and different time points. Correlation between PD-1 and TLR-6 in the late stages of sepsis supports the hypothesis that both may play a synergistic role affecting the histopathological status and functionality of lung tissue. This, also, appears to be mediated by the expression of MyD88 and IRAK. On the contrary, this study did not reveal a significant correlation, neither between TLR-9 and PD-1/PD-L1 nor between NFkB and PD-1/PD-L1.

Sepsis develops over a suppressed immune system in the host and evolves through subsequent hyper- and hypo-inflammatory states, making its therapeutic management extremely challenging for clinicians [46]. In addition, most septic patients quickly reach a state of immunodeficiency and immunosuppression. This is the rationale behind the scientific strategy of developing effective immunosuppression blockers whilst enhancing immune response during sepsis. This has been also supported by the lack of improvement in the mortality of sepsis, despite the development of antibiotics, which suggests the existence of an underlying immune disorder. A possible pathophysiologic pathway of this could be the TLR-mediated PD-1 induction through which bacteria and other pathogens induce the inhibition of the immune response during sepsis. The further elucidation of these pathways and interactions, as provided by this study and other similar or future ones, could be instrumental in the addition of novel therapeutic avenues in the clinician’s armamentarium. For example, monoclonal antibodies against PD-1 and PD-L1 for the treatment of sepsis are under investigation, as are TLR agonists combined with PD-1 inhibitors against solid tumors, based on the hypothesis that combined treatment could enhance the effectiveness of PD-1 blockade [47,48]. Therefore, considering sepsis as a multilevel and progressive disorder and studying the correlation between TLRs and PD-1 expression could be useful and may guide future therapeutic strategies.

As an area of promising future investigation, one may study the relationship between TLR expression as it is related to surfactant protein function and up-regulation. For example, the SPA-4 peptide derived from the C-terminal of TLR-4 has been shown to reduce bacterial burden and decrease the inflammatory response in infected mice.

### Limitations of the Study

The study did have a number of limitations, such as, for example, the inherent physiologic difference in the inflammatory response between mice and humans. Moreover, the CLP model was utilized in the present study despite it not totally representing the pathophysiologic pathways of several other septic conditions, such as, for example, respiratory infections that constitute a significant etiology of sepsis. However, the mouse CLP model represents the most accessible, practical, and adaptable model currently available to researchers. Therefore, it is one of the most utilized, especially considering its affordability. In addition, genetic heterogeneity, supportive care for critically ill patients, the parenteral administration of nutritional supplements, extreme age, and the administration of antibiotics are also crucial parameters that might affect the response to sepsis and were not assessed in the current experimental model. Only male mice were utilized in this study, and although this is a common experimental practice, this could have limited the generalization and applicability of our findings. Another limitation of our study included the restricted time frame and sampling points utilized. We were only able to obtain a picture of the inflammatory response for up to 72 h, which, for example, prevented us from demonstrating the expected reduction in IL-10 levels that occurs. We did this because methodologically, it was believed that a 72 h time frame would be adequate (and is adequate and thus utilized in most reported studies) to demonstrate the initial phase of sepsis response. The study utilized IL-6 and IL-10 as markers of tissue damage and T-cell polarization based on the available literature [49]. Additional markers such as of TNF-alpha, other interleukins, or IFN-y could also have been analyzed, although this would have stretched the available resources with uncertain results. In addition, we followed the principle of reduction as set by Russell and Burch and adapted by all guidelines for the ethical treatment of animals; however, this potentially reduced the amount of available data [50,51]. Considering that the study focused mainly on TLR and PD-1/PD-L1 activation, other pathways/receptors that potentially could act synergistically were not investigated. However, the literature evidence supports this activation mechanism as the most probable. In terms of the histological analysis, flow cytometry or immunohistochemical staining was not performed to demonstrate immune cell (such as neutrophil, macrophage, or lymphocyte) migration due to limited resources and funding, instead utilizing H&E staining, which is a robust, reliable, and affordable method. Furthermore, we were not able to specifically identify which cell type(s) express these molecules, which would have entailed performing mRNA expression analysis on total purified lung tissue, due to budgetary constraints. Similarly, due to a lack of resources, subset isolation and more extensive qPCR tests were not utilized as research strategies; these could have been more revealing of the pathway structure.

## 4. Materials and Methods

### 4.1. Animal Model

In the present study, 60 male C57BL/6J mice (Mus musculus) aged 12–14 weeks were included and sacrificed according to a standardized methodology used in previous protocols [31]. The study was conducted at the Experimental Surgical Center of the Biomedical Research Foundation of the Academy of Athens (BRFAA), which has been approved and accredited by the Prefecture of Attika’s Veterinary Service. The study protocol was approved by the local ethics committee (animal experiment approval number 2838/21, July 2011). The animals were supplied from the available colony of the Experimental Surgical Center of the BRFAA. The body weights of all animals were regularly measured and maintained between 20 and 25 g in order to prevent any adverse conditions that could have affected the septic animal model and possibly result in biased results. The research staff received special training in animal care and handling according to Animal Research Reporting of In Vivo Experiments (ARRIVE) guidelines [52]. The animals were housed in different cages and kept under the following constant environmental conditions: temperature of 18–21 °C, humidity of 40–50%, and artificial day–night cycle of 12:12 h [52]. All animals had unlimited access to food and water using standardized and balanced industrial nutrition. In order to avoid biased results and to sham the statuses of the animals, measurements of the body weights of the animals was regularly applied [31].

### 4.2. Experimental Procedure and Tissue Preparation

The animals were initially randomly separated into six different groups. Each group consisted of ten mice. Three groups were characterized as sham (Sh) groups and the remaining three formed the septic (S) groups. Sepsis in S groups was induced utilizing a cecal ligation and puncture (CLP) model for polymicrobial sepsis, as previously described by Bakopoulos et al. [31] and Rittirsch et al. [32]. Specifically, a midline incision approximately 1 cm in length was performed in each animal’s abdomen and the cecum was carefully exposed so as not to disrupt its vascular supply [52]. The distal one-third of the cecum was ligated with a 3.0 silk suture (Johnson & Johnson, Edinburgh, UK), and by using a 21-gauge needle, a single puncture was performed in the cecum, allowing for the release of fecal material into the peritoneal cavity. It was then placed back into the abdominal cavity and the small laparotomy was approximated in two layers using 4.0 Vicryl sutures (Johnson & Johnson, Edinburgh, UK) [31]. A single puncture was utilized because it had been previously observed, in a pilot setting, that more penetrations resulted in an earlier death for the animals, sometimes even within 24 h [31]. The animals of the Sh groups underwent a sham operation, i.e., no ligation and puncture of the cecum was performed after the entrance into the peritoneal cavity. No bowel content was recognized during sham operations. Both groups were subjected to the same protocol in the terms of resuscitation, analgesic treatment, monitoring, and free access to food and water.

Twenty-four hours after surgery, a group of S animals (S24) and a group of Sh animals (Sh24) were sacrificed. This process was repeated 48 h after surgery for S animals (S48) and Sh animals (Sh48), as well as 72 h after surgery for S animals (S72) and Sh animals (Sh72). Under isoflurane-induced anesthesia, blood samples were drawn by intracardiac aspiration and the animals were euthanized. Body weight was measured before euthanasia. Following euthanasia, the lungs from each mouse were harvested and perfused with ice-cold phosphate buffer solution (PBS), pH 7.4 [31]. The lungs were dissected out into equal pieces and a segment of tissue was formalin-fixed and subsequently paraffin-embedded for histological analysis; the rest of the tissue was rinsed with diethyl pyrocarbonate (DEPC) solution and stored via shock freezing to −80 °C for qRT-PCR analysis [53].

### 4.3. Hematological Analysis

Blood samples were obtained at 24, 48, and 72 h after sepsis induction for the different animal groups depending on the time of euthanasia. White blood cells (WBCs), red blood cell (RBC), and percentages of lymphocytes (LYs) and monocytes (MOs) were analyzed in a blood test analyzer machine (Nihon Kohden, Shinjuku, Tokyo, Japan). Creatinine levels were measured using a biochemical analyzer machine (Chemical 2910 Awareness Technology Inc., Palm City, FL, USA) by enzymatic colorimetric methods. Moreover, interleukin-6 (IL-6) and interleu-kin-10 (IL-10) were measured using Elisa Kits (Quantikine mouse IL-6, IL-10 immunoassay kit; R&D systems, Wiesbaden, Germany).

### 4.4. Histopathological Analysis

The histopathological analysis was performed according to previously described methodology [31]. Ten paraffin sections of 5 μm each were prepared from the animals. Tissue injury and cellular damage was initially assessed by hematoxylin and eosin staining under 40× magnification. Immunohistochemical staining was performed for TLR-5, TLR-6, TLR-9, MYD88, IRAK1, TIRAP, NFkB, PD-1, and PDL-1 on 5 μm deparaffinized sections of formaldehyde-fixed lung tissue using the following rabbit polyclonal antibodies: TLR-5 rabbit polyclonal (Imgenex, Bhubaneswar, India, IMG-580; dilution 5 μg/mL), TLR-6 rabbit polyclonal (Abcam, Cambridge, UK, ab71429; dilution 2 μg/mL), TLR-9 mouse monoclonal (Imgenex, IMG-305A; dilution 2 μg/mL), MYD88 (Santa Cruz. Inc., Dallas, TX, USA, sc-74532; dilution 5 μg/mL), IRAK-1 (Santa Cruz. Inc., sc-5288; dilution 2 μg/mL), TIRAP (GeneTex, Hsinchu City, Taiwan, GTX77618), NFkB (Abcam, ab86299; dilution 2 μg/mL), PD-1 (Biorbyt, Cambridge, UK, orb13641; dilution 2 μg/mL), and PDL1 mouse monoclonal (Abcam, ab238697; dilution 2 μg/mL). Antigen retrieval unmasking was performed via a heat-mediated antigen retrieval method in 0.01 M citric acid (pH 6.0). The ZytoChem Plus HRP polymer anti-mouse/rabbit detection kit (ZYTOMED Systems GmbH, Berlin, Germany) was used for the antibody complex development as described by the manufacturer.

Specifically, 5 μm paraffin sections were deparaffinized by heating to 60 °C, then they were immersed in xylene and subsequently hydrated in a graded series of ethanol aqueous solutions. For antigen retrieval, a citrate buffer (0.01 M, pH 6.0) was used with samples that had been heated for 10 min in an 800 W microwave. A phosphate buffer solution was used for washes after each step of the protocol. The sections were subsequently immersed in a freshly prepared 3% hydrogen peroxide (H_2_O_2_) solution for 10 min in the dark, thus blocking endogenous peroxidase activity. Next, immersion in an Avidin/Biotin complex solution for 15 min in room temperature was performed. Following this, sections were then incubated with the blocking serum, which was supplied by the ZytoChem Plus kit, for 5 min so as to block non-specific staining. Antibodies against TLRs-5, -6, and -9 were diluted by 1:200 in 0.01 mol/L phosphate-buffered saline (PBS) (pH 7.4) and were finally incubated overnight at 4 °C. The sections were then washed 3 times for 5 min using the PBS solution. A specific secondary goat anti-rabbit antibody TRITC, which was diluted (1:200), was incubated at room temperature for 60 min. Sections were washed 3 times for 5 min with PBS. For the nuclei, DAPI counterstaining was utilized. Aqueous medium was used in the cover slip. Finally, analysis and quantification of immunohistochemical sections with DAB color (brown nuclei) was performed by microscopic examination in a Leica DMRA2 microsystem (Leica Microsystems, Wetzlar, Germany) under 40× magnification and photographed using a digital camera, C11440 ORCA flash 4.0 (Hamamatsu, Hamamatsu City, Japan). Computer-assisted morphometry of staining on each image was performed, as opposed to the less accurate semi-quantification by visual scoring, so as to measure the area of each tissue field, the percentages of the background, the percentage of cellular nuclei, and the percentage of expression of each TLR using the ImageJ program (version 1.49C, Wayne Rasband, National Institutes of Health, Bethesda, MD, USA). For the measurement of the relative concentration of the stained molecules, the segmental stained tissue area was expressed as a percentage of the whole tissue area and quantified as %Percentage of Stained Tissue (%PoS).

### 4.5. Real-Time Polymerase Chain Reaction (qRT-PCR)

Total RNA was extracted utilizing the TRIzol reagent according to the manufacturer’s instructions (Life Technologies-Invitrogen, Carlsbad, CA, USA). RT (M-MLV, Reverse transcriptase, Sigma-Aldrich, Burlington, MA, USA) was used to synthesize cDNA and a real-time quantitative polymerase chain reaction was performed by using SYBR Green (Invitrogen, Life Technologies, Carlsbad, CA, USA), following the manufacturer’s established protocol [53,54]. Every sample was run in duplicate and the mean value was used for all further calculations. The 2-Delta Delta CT (2^−ΔΔCT^) method was applied to calculate the relative changes in gene expression. All data were normalized by GAPDH levels and expressed as % relative to shams as previously described [54]. The previously described and reported methodology for data statistical analysis using the 2^−ΔΔCT^ method was utilized for protein expression quantification and to correlate proteins [54].

Table 4 demonstrates the utilized primers that were synthesized by Eurofins Genomic (OriGene Technologies, Inc., Rockville, MD, USA).

### 4.6. Statistical Analysis

One-way analysis of variance (ANOVA) with a repeat method design was used to account for variations within subjects across different conditions and Student’s *t*-test for independent groups, assuming normal distributions of variables, was used for data analysis. Blood data analysis was performed with Tukey’s multiple-comparison test. Correlation between mRNA expression of genes was assessed by Pearson’s correlation. Normalization and log normalization of mRNA expression data were performed using GAPDH levels prior to analysis as per described methodology [54].

Values are expressed here as means ± SDs. Statistically significant differences between the means of the groups were determined and reported using a *p* < 0.05 level of significance. GraphPad Prism version 4.03 (GraphPad Software, Boston, MA, USA) was used to perform statistical and data analysis.

## 5. Conclusions

To the best of our knowledge, this was the first attempt to study the correlation between PD-1/PD-L1, TLRs, and their regulatory molecules in the same septic model. Our study suggested a strong correlation between TLR expression and PD-1/PD-L1 up-regulation in lung tissue during sepsis. This over expression might be related to the histopathological changes in lung tissue during sepsis and to the associated acute lung injury. Certainly, more studies are needed to elucidate the exact mechanisms and molecular pathways. Nevertheless, a combined intervention on TLRs and their inhibitors could suggest a novel therapeutic target for sepsis.

## Figures and Tables

**Table 1:** Blood and biochemical outcomes. Values are expressed as means ± SDs (standard deviations). Statistical significances (*p* < 0.05) between the groups at the same time point are indicated as follows: (a) sham vs. septic, (b) 24S vs. 48S, (c) 48S vs. 72S, and (d) 24S vs. 72S.

	Groups	Sh24	Sh48	Sh72	S24	S48	S72	*p* < 0.05
WBC	Mean	7.66	7.62	7.66	1.74	4.05	6.81	a,b,c,d
(10^3^/mm^3^)	SD	1.64	2.56	1.92	0.43	0.75	1.64	
RBC	Mean	6.21	6.7	6.67	7.94	5.22	2.24	a,b,c,d
(10^6^ µL)	SD	1.53	0.75	1.25	0.87	0.91	0.46	
LY	Mean	79.6	77.5	77.5	64.4	50.8	38.9	a,b,c,d
(%)	SD	7.73	11.41	8.43	4.36	7.85	5.38	
MO	Mean	3.4	3.5	3.1	3.8	5.7	8.1	a,b,c,d
(%)	SD	1.35	1.71	1.52	1.22	0.82	0.99	
IL-6	Mean	14.75	14.76	15.11	1387.1	2881.2	3409.01	a,b,c,d
(mg/dL)	SD	3.53	3.22	2.22	268.9	486.8	415.7	
IL-10	Mean	7.06	6.66	7.74	182.2	339.6	990.7	a,b,c,d
(mg/dL)	SD	1.43	1.59	2.38	42.01	86.92	156.4	

**Table 2:** Quantification of antibody histochemistry expression in sepsis at different experimental time points. Numbers represent mean percentages of stained area—cells (expression) %PoS. SD stands for standard deviation of the mean.

Antibody Expression (%PoS) vs. Time		S24	S48	S72
PD-1	mean	6.670	8.692	11.13
	SD	0.733	0.619	0.847
PDL-1	mean	7.957	10.16	12.85
	SD	1.391	1.546	1.733
TLR5	mean	1.462	3.045	4.522
	SD	0.514	0.489	0.814
TLR6	mean	5.992	5.997	11.31
	SD	1.330	0.970	0.918
TLR9	mean	14.50	18.39	23.92
	SD	2.168	1.488	1.855
MYD88	mean	3.072	6.307	8.537
	SD	1.046	0.913	1.533
IRAK1	mean	3.767	7.167	8.455
	SD	1.346	1.110	1.284
TIRAP	mean	5.385	8.528	12.19
	SD	0.873	1.085	1.270
NFkB	mean	2.752	4.815	9.998
	SD	1.059	1.057	1.240

**Table 3:** Correlation between mRNA expressions of genes. White boxes show no statistical significance; green boxes show statistical significance for different genes and different groups; blue boxes show statistical significance for different genes and same groups.

	S24-PD1	S48-PD1	S72-PD1	S24-PDL1	S48-PDL1	S72-PDL1	S24-TLR5	S48-TLR5	S72-TLR5	S24-TLR6	S48-TLR6	S72-TLR6	S24-TLR9	S48-TLR9	S72-TLR9	S24-MYD88	S48-MYD88	S72-MYD88	S24-IRAK1	S48-IRAK1	S72-IRAK1	S24-TIRAP	S48-TIRAP	S72-TIRAP	S24-NFkB	S48-NFkB	S72-NFkB
S24-PD1																											
S48-PD1	0.005																										
S72-PD1	0.743	0.722																									
S24-PDL1	0.003	0.0009	0.688																								
S48-PDL1	0.001	0.004	0.599	0.004																							
S72-PDL1	0.0007	0.0007	0.754	0.0001	0.0007																						
S24-TLR5	0.955	0.652	0.482	0.702	0.456	0.769																					
S48-TLR5	0.994	0.804	0.653	0.48	0.623	0.56	0.24																				
S72-TLR5	0.730	0.948	0.754	0.294	0.409	0.381	0.036	0.142																			
S24-TLR6	0.438	0.459	0.957	0.648	0.671	0.656	0.048	0.942	0.064																		
S48-TLR6	0.007	0.343	0.8	0.123	0.109	0.086	0.256	0.826	0.957	0.146																	
S72-TLR6	0.031	0.05	0.711	0.003	0.049	0.003	0.480	0.319	0.606	0.166	0.04																
S24-TLR9	0.831	0.341	0.582	0.495	0.148	0.443	0.201	0.523	0.345	0.511	0.406	0.733															
S48-TLR9	0.074	0.523	0.892	0.154	0.288	0.108	0.354	0.535	0.767	0.319	0.005	0.023	0.212														
S72-TLR9	0.761	0.662	0.544	0.742	0.924	0.987	0.65	0.272	0.076	0.776	0.94	0.669	0.422	0.596													
S24-MYD88	0.068	0.02	0.541	0.0008	0.133	0.007	0.686	0.326	0.313	0.576	0.265	0.01	0.918	0.147	0.699												
S48-MYD88	0.195	0.045	0.642	0.049	0.062	0.031	0.443	0.65	0.404	0.295	0.99	0.365	0.13	0.933	0.549	0.257											
S72-MYD88	0.002	0.001	0.796	0.0001	0.0009	0.0001	0.471	0.319	0.193	0.842	0.160	0.007	0.311	0.182	0.718	0.005	0.042										
S24-IRAK	0.038	0.014	0.284	0.0302	0.018	0.022	0.815	0.23	0.803	0.983	0.364	0.292	0.288	0.868	0.653	0.214	0.003	0.046									
S48-IRAK	0.061	0.017	0.361	0.0253	0.233	0.034	0.266	0.925	0.534	0.144	0.188	0.009	0.948	0.283	0.953	0.037	0.366	0.068	0.204								
S72-IRAK	0.061	0.411	0.847	0.187	0.028	0.064	0.951	0.836	0.325	0.686	0.106	0.18	0.406	0.092	0.74	0.802	0.127	0.09	0.241	0.772							
S24-TIRAP	0.972	0.544	0.774	0.513	0.488	0.466	0.362	0.008	0.58	0.835	0.632	0.393	0.599	0.407	0.541	0.395	0.744	0.373	0.58	0.882	0.627						
S48-TIRAP	0.503	0.737	0.222	0.812	0.713	0.949	0.722	0.087	0.531	0.844	0.418	0.746	0.029	0.684	0.442	0.427	0.905	0.872	0.47	0.584	0.367	0.139					
S72-TIRAP	0.676	0.531	0.793	0.447	0.807	0.756	0.186	0.289	0.199	0.766	0.279	0.811	0.113	0.133	0.028	0.31	0.698	0.477	0.698	0.845	0.253	0.84	0.554				
S24-NFkB	0.396	0.761	0.657	0.788	0.785	0.61	0.232	0.602	0.9	0.379	0.045	0.178	0.155	0.0003	0.444	0.723	0.527	0.784	0.47	0.665	0.152	0.45	0.98	0.021			
S48-NFkB	0.628	0.875	0.455	0.671	0.887	0.661	0.292	0.175	0.566	0.122	0.281	0.135	0.127	0.017	0.577	0.3159	0.274	0.74	0.193	0.273	0.939	0.159	0.966	0.28	0.008		
72-NFkB	0.857	0.572	0.918	0.627	0.546	0.748	0.472	0.672	0.361	0.792	0.322	0.54	0.1	0.18	0.284	0.439	0.654	0.721	0.54	0.827	0.785	0.694	0.522	0.801	0.328	0.374	

**Table 4:** Primer sequences.

Gene	Accession No.	Forward Primer	Reverse Primer
*Tlr-5*	NM_016928	TCCTGACCAGAGCACATTTGCC	CCTTCAGTGTCCCAAACAGTCG
*Tlr-6*	NM_001359180	GTGAGGATGCTGTGTCAGTGGAG	CCAGGCAGAATCATGCTCACTG
*Tlr-9*	NM_031178	GCTGTCAATGGCTCTCAGTTCC	CCTGCAACTGTGGTAGCTCACT
*Myd88*	NM_010851	ACCTGTGTCTGGTCCATTGCCA	GCTGAGTGCAAACTTGGTCTGG
*Irak1*	NM_008363	GGACTTCCACAGTTCGAGGTAC	GGTCTTTGCACCTTGTGTCCTC
*Tirap*	NM_054096	ATCTCCCAGGAAAGCCACCTCT	GGTAGGTGACATTCCTGAACTGC
*Nfkb*	NM_008689	GAAATTCCTGATCCAGACAAAAAC	ATCACTTCAATGGCCTCTGTGTAG
*Pdcd1* (PD-1)	NM_008798	CGGTTTCAAGGCATGGTCATTGG	TCAGAGTGTCGTCCTTGCTTCC
*Cd274* (PDL-1)	NM_021893	TGCGGACTACAAGCGAATCACG	CTCAGCTTCTGGATAACCCTCG
*Gapdh*	NM_001289726	CATCACTGCCACCCAGAAGACTG	ATGCCAGTGAGCTTCCCGTTCAG

## Data Availability

The data presented in this study are available on request from the corresponding author. The data are, however, not publicly available.
